# *TEMPy*: a Python library for assessment of three-dimensional electron microscopy density fits

**DOI:** 10.1107/S1600576715010092

**Published:** 2015-06-27

**Authors:** Irene Farabella, Daven Vasishtan, Agnel Praveen Joseph, Arun Prasad Pandurangan, Harpal Sahota, Maya Topf

**Affiliations:** a Institute of Structural and Molecular Biology, Department of Biological Sciences, Birkbeck, University of London, Malet street, London WC1E 7HX, UK; b Oxford Particle Imaging Centre, Division of Structural Biology, Wellcome Trust Centre for Human Genetics, University of Oxford, Oxford OX3 7BN, UK; c Scientific Computing Department, Science and Technology Facilities Council, Research Complex at Harwell, Didcot, Oxon OX11 0QX, UK

**Keywords:** three-dimensional electron microscopy, macromolecular structures, model assessment

## Abstract

Three-dimensional electron microscopy is currently one of the most promising techniques used to study macromolecular assemblies. Rigid and flexible fitting of atomic models into density maps is often essential to gain further insights into the assemblies they represent. Currently, tools that facilitate the assessment of fitted atomic models and maps are needed. *TEMPy* (template and electron microscopy comparison using Python) is a toolkit designed for this purpose. The library includes a set of methods to assess density fits in intermediate-to-low resolution maps, both globally and locally. It also provides procedures for single-fit assessment, ensemble generation of fits, clustering, and multiple and consensus scoring, as well as plots and output files for visualization purposes to help the user in analysing rigid and flexible fits. The modular nature of *TEMPy* helps the integration of scoring and assessment of fits into large pipelines, making it a tool suitable for both novice and expert structural biologists.

## Abstract 2

*TEMPy* is an object-oriented Python library that provides the means to validate density fits in electron microscopy reconstructions. This article highlights several features of particular interest for this purpose and includes some customized examples.

## 1. Introduction   

The integration of data derived from a variety of biophysical techniques at multiple levels of resolution, such as electron microscopy (EM), small-angle X-ray scattering, X-ray crystallography or nuclear magnetic resonance (NMR) spectroscopy, is becoming common in the structural determination of large macromolecular assemblies (Ward *et al.*, 2013 ▸; Lander *et al.*, 2012 ▸; Karaca & Bonvin, 2013 ▸). This combination, often aided by computational methods, allows scientists to gain further insights into the macromolecular assemblies they study. An example is the fitting of atomic structures into three-dimensional EM (3D EM) density maps. At the beginning of 2015, out of 2770 maps in the Electron Microscopy Data Bank (spanning a wide range of resolutions, mostly between 5 and 20 Å; Milne *et al.*, 2013 ▸; http://www.ebi.ac.uk/pdbe/emdb), a total of 653 entries were linked to fitted atomic models in the Protein Data Bank (PDB; http://www.rcsb.org/pdb). Density fitting depends on the amount of information in the experimental data, the accuracy of the starting model, the complexity of their representation and the scoring function representing the goodness-of-fit (Henderson *et al.*, 2012 ▸; Thalassinos *et al.*, 2013 ▸). Currently, most 3D EM density maps do not allow for an unambiguous placement of individual atoms. The number of parameters to be solved is experimentally underdetermined and false-positive solutions are likely. To reduce the number of parameters, ‘rigid’ fitting is often performed on a given structural unit (*e.g.* a whole protein or a domain), that is, without changing the relative positions of the individual atoms within the unit. A global search is performed in six degrees of freedom to find the position and orientation of the atomic model in the map that gives the best fit between the two (Esquivel-Rodríguez & Kihara, 2013 ▸; Thalassinos *et al.*, 2013 ▸). However, rigid fitting can also be performed locally, if prior knowledge about the approximate position of the model in the map exists (Topf *et al.*, 2005 ▸; Goddard *et al.*, 2007 ▸). Furthermore, 3D EM maps often represent conformational states that differ from the initial conformation of the atomic model (Thalassinos *et al.*, 2013 ▸; Villa & Lasker, 2014 ▸). In such cases, to gain insight into the dynamic properties of the structure, flexible fitting is applied, by changing the conformation of the initial atomic model while improving the goodness-of-fit. However, here too, additional constraints have to be applied to reduce the probability of overfitting (Topf *et al.*, 2008 ▸). Increasingly, 3D EM density maps are achieving high resolution (∼3–4 Å), allowing *de novo* models to be generated and the use of tools adapted from the X-ray crystallography field (Brown *et al.*, 2015 ▸; Wang *et al.*, 2014 ▸).

In addition to identifying an appropriate fitting method, it is important to assess the accuracy of the fitted model (the difference from the true structure) as well as its precision (the variability from other models consistent with the data that score similarly) (Alber *et al.*, 2008 ▸). Many tools exist for the assessment of structural models against geometric criteria, such as *MolProbity* (Chen *et al.*, 2010 ▸) and *WHAT_CHECK* (Hooft *et al.*, 1996 ▸). Methods and tools to assess the quality of a model in the context of 3D EM maps are less common, although it is becoming clear that such validation approaches are needed (Henderson *et al.*, 2012 ▸; Ludtke *et al.*, 2012 ▸). One approach is the use of confidence intervals and docking precision estimates in global rigid body fitting (Volkmann, 2009 ▸). This approach was used to assess the fit of the first 559 residues of the 2.5 MDa ryanodine receptor (RyR1) crystal structure within the cryo EM map of the entire complex at 9.6 Å (Tung *et al.*, 2010 ▸; Garzón *et al.*, 2007 ▸; Wriggers & Birmanns, 2001 ▸), which has recently been confirmed on the basis of higher resolution maps (Zalk *et al.*, 2015 ▸; Yan *et al.*, 2015 ▸). This higher resolution model is different from an earlier fit of a comparative model into the same map, which resulted from a local fitting procedure, relying on antibody labelling that constrained the sampling to an incorrect region of the map (Serysheva *et al.*, 2008 ▸). Another validation approach that has been proven useful is the selection of the best-fitting model relative to alternative fits (Vasishtan & Topf, 2011 ▸; Wriggers & Birmanns, 2001 ▸; Roseman, 2000 ▸). For instance, alternative models generated by protein structure prediction methods can be assessed by selecting the model that fits best into the map (Topf *et al.*, 2005 ▸). Such an approach has been used in structure characterization of macromolecular assemblies, including eukaryotic ribosomes (Chandramouli *et al.*, 2008 ▸; Taylor *et al.*, 2009 ▸) and herpesviruses capsids (Baker *et al.*, 2005 ▸). Interestingly, for structural characterization of a tobacco mosaic virus map at 4.4 Å resolution, four models generated by real-space mol­ecular dynamics were suggested in order to provide a better representation of the data (Sachse *et al.*, 2007 ▸). In the context of lower resolution maps it has been suggested to use much larger ensembles to describe coordinate uncertainty in certain regions of the map (Lukoyanova *et al.*, 2015 ▸; Goulet *et al.*, 2014 ▸). For example, for the three intermediates of a membrane attack complex/perforin-like protein (pleurotolysin) at resolutions of 14–17 Å, 20 models generated by angular sweeps were suggested (and deposited in the PDB; Lukoyanova *et al.*, 2015 ▸).

Another validation approach, in the context of flexible fitting, is the use of multiple flexible fitting methods in order to reach a consensus fit and measure the local fit reliability using root-mean-square fluctuations and local correlation (Ahmed *et al.*, 2012 ▸; Ahmed & Tama, 2013 ▸; Pandurangan *et al.*, 2014 ▸). This approach has helped in the refinement of the coxsackievirus A7 capsid in subnanometre resolution cryo EM maps representing two conformations (Pandurangan *et al.*, 2014 ▸).

Finally, cross-validation methods have also been used to identify the optimally fitted model of cyclic nucleotide-modulated ion channels (MloK1) in a large density map at 16 Å resolution (Schröder *et al.*, 2010 ▸; Kowal *et al.*, 2014 ▸) and to validate an all-atom *de novo* model of the brome mosaic virus obtained from a 3.8 Å resolution map (Wang *et al.*, 2014 ▸; DiMaio *et al.*, 2013 ▸).

Previously, we proposed that a useful way of assessing models is the use of a variety of goodness-of-fit scores (Vasishtan & Topf, 2011 ▸). Although a number of scoring methods have been developed, different scores have specific advantages. Thus, their combination could be proven useful in different scenarios. These include the most commonly used cross-correlation coefficient and its variations (*e.g.* Laplacian-filtered cross-correlation coefficient; Wriggers & Chacón, 2001 ▸), a mutual information-based score, and edge-based scores (Vasishtan & Topf, 2011 ▸). The scoring in most programs for rigid and flexible fitting (which are available either independently or as part of image processing packages; Heymann, 2001 ▸; Ludtke *et al.*, 1999 ▸; Villa & Lasker, 2014 ▸) is based primarily on cross-correlation methods. However, there are currently no tools that allow the assessment of fit quality using a large selection of scoring methods either independently or as a means of consensus in the same platform.

Here, we implement such a platform, called *TEMPy* (template and EM comparison using Python). The software is useful for density map and atomic structure processing and for fit assessment (model-to-map and map-to-map), especially in the intermediate-to-low resolution range. It provides a selection of scoring functions that allow the user to assess the reliability of density fits, which can be used in conjunction with ensemble generation of alternative fits and clustering, as well as consensus scoring. Additionally, the capability to provide local fit assessment based on any user-defined structure segment (*e.g.* a protein domain or a secondary structure element) can be useful in flexible fitting, particularly at subnanometre resolution (Pandurangan & Topf, 2012 ▸). *TEMPy* can also provide plots and output files for visualization purposes that can further help the user in analysing the results. The software has already been used for fit assessment of multiple conformers of coxsackievirus A7 (Pandurangan *et al.*, 2014 ▸), microtubule-bound kinesins 1 and 3 (Atherton *et al.*, 2014 ▸), and pleurotolysin (Lukoyanova *et al.*, 2015 ▸).

## 2. Software design and implementation   

*TEMPy* is a cross-platform package implemented in the Python programming language, which uses the *NumPy* and *SciPy* libraries (Jones *et al.*, 2001 ▸), making it computationally efficient. Additionally, it uses *Biopython* for handling atomic coordinate files (Hamelryck & Manderick, 2003 ▸). The program is flexible, allowing the users to build customized functions. The modular organization of the software supports its integration into large modular pipelines or into larger software suites, such as *CCP-EM* (Wood *et al.*, 2015 ▸).

### 2.1. Input/output   

*TEMPy* currently supports reading and writing of density maps in CCP4/MRC format and atomic structures in PDB or mmCIF format. It can parse subsets of atomic structures (rigid bodies) as simple text files (for example *RIBFIND* output files; Pandurangan & Topf, 2012 ▸). It can also generate *Chimera* (Pettersen *et al.*, 2004 ▸) input files for visualization of fits, as well as high-quality plots using the Python library *Matplotlib* (Hunter, 2007 ▸), to help the user with the interpretation of the results using a colour gradient.

### 2.2. Core modules   

*TEMPy* consists of a number of core Python modules, including the Map module (*EMMap.py*) for processing density maps; the Structure module (*ProtRep.py*) for processing atomic structures; the Structure Blurrer module (*StructureBlurrer.py*) for creating density maps from atomic structures; the Scoring Functions module (*ScoringFunctions.py*) that contains a variety of methods for scoring density fits; and the Ensemble Generation module (*EnsembleGeneration.py*) for generating ensembles of fits. The program can also load alternative fits or ensembles generated by other programs based on approaches such as density fitting combined with molecular docking (Lasker *et al.*, 2010 ▸; Esquivel-Rodríguez & Kihara, 2012 ▸), normal mode analysis (Tama *et al.*, 2002 ▸), molecular dynamics (Trabuco *et al.*, 2008 ▸), comparative modelling (Topf *et al.*, 2005 ▸) and loop modelling (Goulet *et al.*, 2014 ▸). These ensembles can be analysed using the Clustering module (*Cluster.py*) and the Consensus Scoring module (*Consensus.py*), which can be useful in estimating precision and, in some cases, accuracy.

### 2.3. Algorithms   

#### 2.3.1. Scoring functions   

*TEMPy* offers a selection of scoring methods for the assessment of fit quality on a single platform (Table 1 ▸). The cross-correlation coefficient (CCC) is expressed by the following formula (Roseman, 2000 ▸):
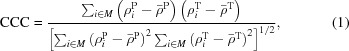
where *M* represents all the voxels in the density grid of the map target, 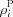 and 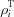 represent the intensities at points *i* in the probe map and target map, respectively, and 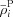 and 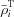 are the respective mean intensities. Different variations on the CCC, such as Laplacian-filtered CCC (LAP) (Wriggers & Chacon, 2001 ▸) and the segment-based cross correlation (SCCC) (Pandurangan *et al.*, 2014 ▸), are also implemented.

The mutual information score (MI) is an entropy-based concept given by the relative entropy between the joint distribution *p*(*x*, *y*) and the product distribution *p*(*x*)*p*(*y*):
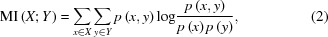
where *X* and *Y* correspond to the density values of the voxels in the probe and target maps. *p*(*x*) and *p*(*y*) are given by the percentage of voxels with density values equal to *x* and *y*, respectively. *p*(*x*, *y*) is given by the percentage of aligned voxels with value *x* in the probe map and *y* in the target map. Since the density values in an EM map take a wide range of values and are typically noisy, it is necessary to bin the map into a limited number of values (typically 20; Shatsky *et al.*, 2009 ▸). A segment-based variation of this score (SMI) is also available in *TEMPy* and is implemented in a manner similar to the SCCC score (Pandurangan *et al.*, 2014 ▸).

The envelope score (ENV) attempts to describe how much of the density map is filled with atoms and penalizes both protrusions from the surface (‘envelope’) and empty spaces in the map. First, all the pixels in the target map are given binary values, based on whether they are higher or lower than a given density threshold. Then, for each atom in the probe structure, the nearest density point in the target map is found and is down- or up-weighted taking into account the binary values of the target map. The sum of all of these values gives the ENV score, which can take any integer value, with the largest values denoting the best fits.

The normal vector score and Chamfer distance are both based on the comparison of the surfaces of the atomic structure and EM map (Vasishtan & Topf, 2011 ▸).

The normal vector (NV) score is a calculation of the difference in angle between the normal vectors of the surfaces of the target and probe maps, in which the vectors are calculated using a variation of the method developed in the *3SOM* algorithm (Ceulemans & Russell, 2004 ▸). The NV score gives non-negative output values and it is expressed as
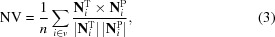
where *n* is the number of normal vectors calculated in the target map, 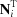 and 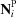 are the normal vectors of the target and probe map, respectively, at point *i* on the surface, and *v* is the set of surface points within the volume threshold. The vertical bars denote the vector magnitude. The score is ranged between 0 and π, where 0 is the best score, *i.e.* there is no difference in the direction of all corresponding normal vectors between the target and probe maps.

The Chamfer distance (CD) is a pattern matching score used successfully in video tracking (Knossow *et al.*, 2007 ▸; Chen *et al.*, 2007 ▸) that has been used recently for assessing 3D EM fits (Vasishtan & Topf, 2011 ▸). The CD between two sets of points, *X* and *Y*, on the surfaces of the target and probe maps, is given by calculating the distance of every point in *X* from its nearest neighbour in *Y* and taking the average of all these values. It is expressed as
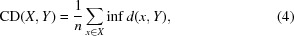
where *n* is the number of points of *X*, 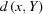 is the set of Euclidean distances between *x* and every point in *Y*, and inf is the infimum. The CD score, like the NV score, gives non-negative values. Zero is the best score, given when all surface voxels in the probe map are perfectly superimposed on the surface voxels in the target map.

Previously, we have used a volume-based threshold method to define the surface (Vasishtan & Topf, 2011 ▸). Here, to improve surface detection within this threshold we implement the Sobel filter (using *Scipy*) (Duda & Hart, 1973 ▸), which has been used in image processing (Pinidiyaarachchi *et al.*, 2009 ▸; Wahlby *et al.*, 2004 ▸). It approximates the gradient of voxel density by convolution of the filter kernel along the axis. The filter kernel consists of averaging (*S*_a_) and differentiation (*H*_a_) kernels:
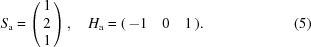
The convolution filter kernels along the three axis directions are separable as
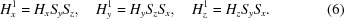
The filtered map A^1^ is obtained as
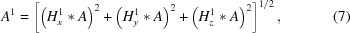
where *A* is the original map and * is the convolution operator.

#### 2.3.2. Clustering   

*TEMPy* provides a procedure for clustering different sets of density fits to identify the best fits (using the Clustering module), for example, by hierarchical root-mean-square deviation (RMSD) clustering. The analysis is accompanied by plots and output files that are readable in *Chimera*, allowing the visualization of the top-scoring fits coloured by clusters in the context of the map (*Plot.py*). This approach helps the user to decide in a more systematic and objective fashion if any one of the fits stands out. In cases where it is not possible to identify a single accurate and/or precise fit (as is often the case in low resolution EM density maps), to better represent the experimental data one could suggest multiple solutions based on the set of good-scoring fits. The variability among this set of solutions represents the precision of the suggested model and/or the lower bound on its accuracy (Schneidman-Duhovny *et al.*, 2014 ▸).

#### 2.3.3. Consensus Scoring   

*TEMPy* provides a consensus scoring (using the Consensus module). We implemented this option based on the Borda count: a preference method-based voting system that has been used to compute consensus in networks (Brush *et al.*, 2013 ▸) and in ligand-based docking (Ahmed *et al.*, 2014 ▸). Each fit in an ensemble of *N* fits is ranked on the basis of a list of *S* different scores (*S* > 1, any given combination of scores can be chosen). Given a score *i*, a ranking score (*r*) is assigned to each fit according to its positional order in the ensemble. The Borda score is defined as
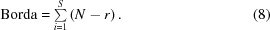

*TEMPy* also offers the possibility to visualize the ranked fits (with the support of a colour-coded interpretation of the results), which can help the user to interpret the consensus among the scoring metrics chosen.

## 3. Application examples   

**Figure 1:**
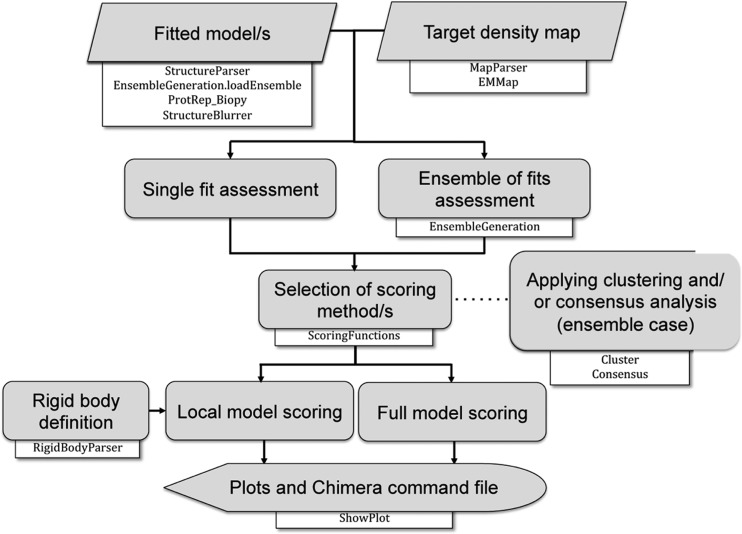
Workflow in *TEMPy* for assessing atomic models fitted in 3D EM density maps.

*TEMPy* provides procedures for single-fit assessment, ensemble generation of fits, clustering, multiple scoring and consensus scoring (Fig. 1 ▸). In principle, any type of set of fits (model-to-map and map-to-map) can be assessed both globally and/or locally in a map using the entire structure or just parts of it. Below we describe a set of test cases to highlight some of these capabilities.

### 3.1. Assessment of ensemble of fits   

Different strategies to detect the most appropriate solution within a set of alternative fits, such as hierarchical clustering and consensus scoring protocols, are implemented in *TEMPy*. To show how these strategies can be employed to identify a fit that stands out among a set of alternative solutions, we provide four different types of examples that cover a fair range of expected fitting scenarios in maps at intermediate-to-low resolution. In the first two examples we used *TEMPy* to generate an ensemble of alternative fits around a given initial fit (local search) using 12 simulated maps (Example 1, §3.1.1) and one experimental map (Example 2, §3.1.2). In the third and fourth examples we used as an input to *TEMPy* ensembles generated elsewhere: by collecting different conformations (from the PDB) of a given initial fit into an experimental map (Example 3, §3.1.3); and from a global search performed by another fitting program (Example 4, §3.1.4). In each case, we used the Ensemble module, Scoring Function module, Clustering module, and/or Consensus and Plotting modules for assessment. The snippet of code in Fig. S1A in the supporting information shows a few Python code lines that are needed to generate an ensemble of fits, rank them on the basis of a chosen score, hierarchically cluster them on the basis of Cα-RMSD and then visualize the cluster dendrograms of the fits.

#### 3.1.1. Simulated benchmark: assessment of a local search ensemble and score performance   

A total of 12 maps were simulated at 5, 10, 15 and 20 Å resolution from three known X-ray structures: the ligand-free glutamine-binding protein (PDB code 1ggg; Hsiao *et al.*,1996 ▸); the ligand-bound maltodextrin binding protein (PDB code 1anf; Quiocho *et al.*, 1997 ▸); and the ligand-free d-ribose-binding protein (PDB code 1urp; Björkman & Mowbray, 1998 ▸). The maps were produced with the *molmap* command in *Chimera* (Goddard *et al.*, 2007 ▸) using the default sigma factor of 0.225 (setting the maximal grid spacing to 3.5 Å per pixel). For each example a random ensemble of 200 alternative fits was generated with *TEMPy* (0 ≤ Δ*T* ≤ 10 Å and 0 ≤ ΔΦ ≤ 60°).

All fits were scored using four different scores: CCC, MI and NV score, with and without the Sobel filter (NV-S, applied to any densities above the threshold). If the best fits are similar, different scoring methods will typically result in a slightly different ranking. Here, we show how clustering those fits can guide the user to identify the best fit. The 20 top-scoring fits based on each score were hierarchically clustered by Cα-RMSD (using the mean Cα-RMSD of the top 20 fits for each score as a cutoff). Examination of the resulting clusters underlines the performance of each score (*i.e.* the separation between the top fit and the alternative ones) (Figs. S2–S4).

As expected, the starting fit (model 0, which was used to simulate the map) is highlighted by all four scores to be the top-scoring fit within the top-scoring non-singleton cluster. Only in the case of 1urp does some ambiguity arise between model 0 and an alternative fit (model 154) at low resolutions (15 and 20 Å) for all four scores and at higher resolutions (5 and 10 Å) for CCC (Fig. S4). However, the Cα-RMSD between the models is very small (0.32 Å). At 20 Å resolution, using the Sobel filter with the NV score (NV-S) improves the discrimination between the top fits for both 1anf and 1urp. Independent of the target map resolution, in all the test cases the MI score allows better discrimination between alternative solutions.

To show the usability of the consensus approach in the context of EM fits we used the three simulated test cases presented above. Each of the 200 random alternative fits was scored using CCC, MI and NV-S (because of the improved performance over the NV score). The Borda score was then used to re-rank the ensemble of alternative solutions (Tables S1–S12). In the case of 1urp, the ambiguity in the top-ranking solution (due to NV-S ranking model 0 second) is overcome by re-ranking with the Borda score.

#### 3.1.2. Experimental benchmark: assessment of a local search ensemble and score performance   

**Figure 2:**
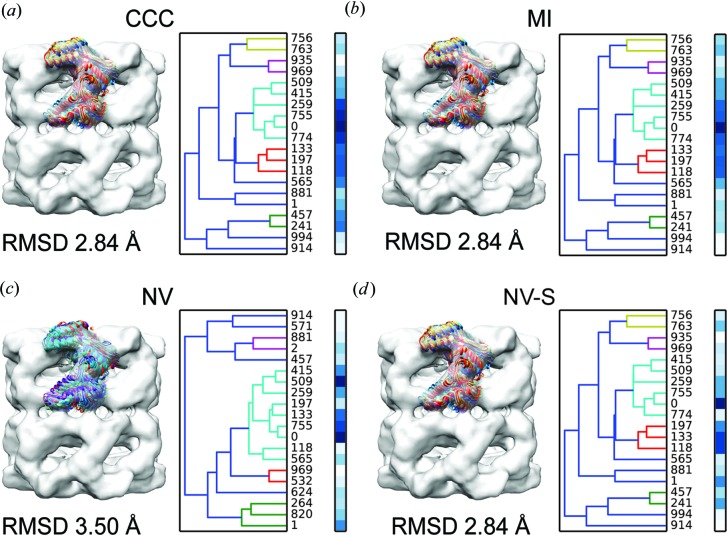
Clustering an ensemble of rigid fits. The 20 top-scoring fits of a single GroEL subunit (PDB code 1oel; Braig *et al.*, 1995 ▸) within the 11.5 Å resolution density map of GroEL (EMD code 1080; Ludtke *et al.*, 2001 ▸) (in grey) are shown based on four different scores: CCC (*a*), MI (*b*), NV (*c*) and NV with Sobel filter (NV-S) (*d*). Left column: the fitted models are shown in the context of the map. Right column: the cluster dendrograms of the fits. The colour bars represent the score of each fit from white (lowest score) to blue (best score). Each cluster is coloured differently and the average Cα-RMSD value is reported below.

We applied the ensemble clustering approach to an experimental case using the X-ray structure of the bacterial chaperonin apo-GroEL (PDB code 1oel; Braig *et al.*, 1995 ▸) and a cryo EM density map of apo-GroEL at 11.5 Å resolution (EMD code 1080; Ludtke *et al.*, 2001 ▸). First, the structure was fitted using the cross-correlation score implemented in *Chimera*’s *fit_in_map* tool (Goddard *et al.*, 2007 ▸). Then, the density map was segmented around a single subunit with a large box using *Chimera*. Next, a random ensemble of 1000 alternative fits was generated with *TEMPy* using 0 ≤ Δ*T* ≤ 5 Å and 0 ≤ ΔΦ ≤ 60° to explore the immediate neighbourhood of fits. The fits were then scored as before using CCC, MI, NV and NV-S. The 20 top-scoring fits based on each score were hierarchically clustered by Cα-RMSD (Fig. 2 ▸). The analysis of the 20 top-scoring fits based on the Cα-RMSD clustering resulted in a similar trend to the simulated data. The starting *Chimera* fit is the top-scoring fit within the top-scoring non-singleton cluster. The MI is again the most discriminatory score and the NV-S more discriminatory than the NV. Here too, we applied the consensus scoring approach. Calculating the Borda score over 1000 alternative fits using CCC, MI and NV-S clearly resulted in model 0 as the best model (Table S13).

#### 3.1.3. Assessment of an ensemble of conformations from the PDB   

**Figure 3:**
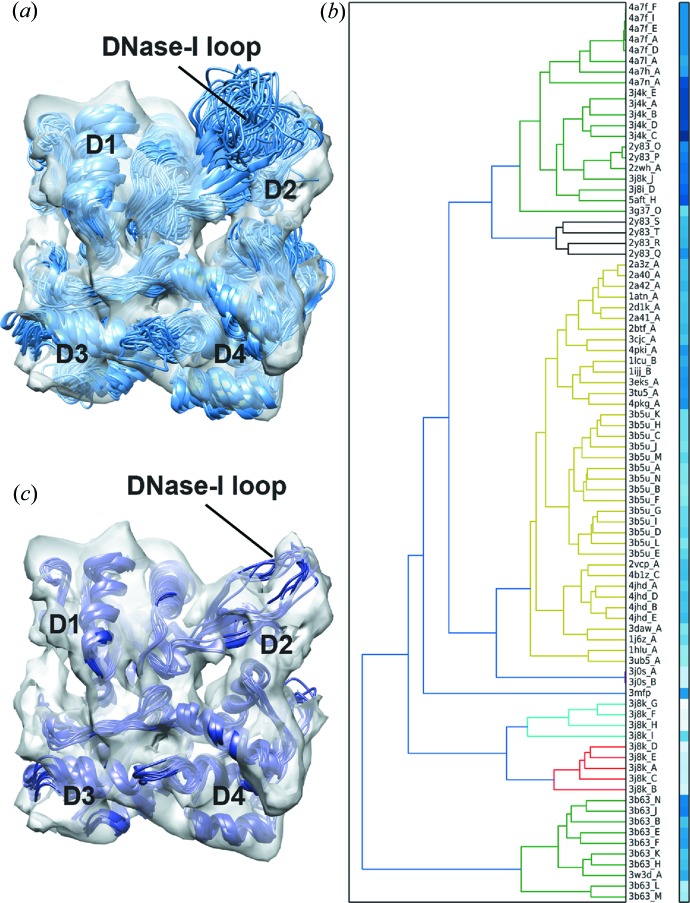
Assessment of an ensemble of conformations from the PDB. (*a*) An ensemble generated from 84 different actin structures (taken from the PDB) is shown (in light blue) within an actin monomer density map [segmented from an 8.9 Å resolution actin filament (EMD code 1990; Behrmann *et al.*, 2012 ▸), in grey]. (*b*) The cluster dendrogram of the fits is shown alongside, with the colour bars representing the CCC score of each fit (from white to blue, low to high score). Each cluster is coloured differently. (*c*) The top 10% scoring fits of the actin monomer from (*a*), selected on the basis of the CCC and MI ranking (data not shown), are shown (in dark blue) within an actin monomer density map [as in (*a*)]. The subdomains 1–4 (D1, D2, D3 and D4, respectively) and the DNase-I loop are labelled.

Using an ensemble generated from actin PDB structures, we applied the ensemble clustering approach to identify a model that best fits into the actin filament map at 8.9 Å resolution (EMD code 1990; Behrmann *et al.*, 2012 ▸). We generated the ensemble based on sequence and structure similarity to an F-actin subunit model (PDB code 3mfp; Fujii *et al.*, 2010 ▸) *via* the *DALI* server (Holm & Rosenström, 2010 ▸), using the following criteria: sequence id > 90%, all-against-all Cα-RMSD ≤ 3.5 Å, removal of incomplete structures and use of a single representative for identical chains. This resulted in 84 structures in total. An actin subunit consists of four subdomains (D1–D4) that are arranged by twist-and-scissors rotation angle (Cong *et al.*, 2008 ▸). Although the overall organization is similar within the structures in the ensemble, RMSD analysis with *TEMPy* showed that differences occur between the subdomains, with the most prominent difference occurring in subdomain D2 (the mean Cα-RMSDs are D1 2.1 Å, D2 4.8 Å, D3 2.1 Å and D4 2.5 Å). Each actin subunit in the ensemble was rigidly fitted into the actin filament map, which was first segmented around a single subunit using *Chimera* (Fig. 3 ▸ *a*). The obtained fitted ensemble was then used as an input into *TEMPy* and scored using the ensemble clustering protocol with CCC and MI. With both scores, several actin structures fit equally well in the map. Hierarchical Cα-RMSD clustering analysis on the complete ensemble resulted in the majority of these top-scoring fits belonging to the same non-singleton cluster [Fig. 3 ▸(*b*) for the CCC analysis]. To better represent the heterogeneity of the experimental map we chose the top 10% scoring fits based on each score (resulting in a total of ten fits) (Fig. 3 ▸ *c*).

#### 3.1.4. Simulated test case: assessment of a global search ensemble   

**Figure 4:**
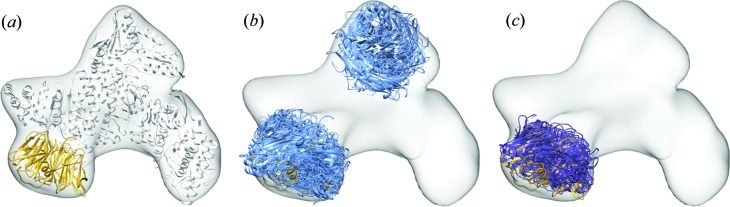
Assessment of a global search ensemble. (*a*) The X-ray atomic structure of the Arp2/3 seven-subunit complex with ATP and Ca^2+^ (PDB code 1tyq; Nolen *et al.*, 2004 ▸) is shown (in light grey) within a corresponding 20 Å resolution simulated map (in grey), with chain C (the seven-bladed beta-propeller ARCP1 subunit) highlighted (in yellow). (*b*) The top-ten-ranked solutions resulting from global fitting of chain C within the 20 Å resolution map based on *ADP_EM* (in light blue) are shown within the density map (in grey). The native chain C is shown as reference in yellow [as in (*a*)]. (*c*) The top-ten-scoring fits (in dark blue) based on re-ranking of the 100 *ADP-EM* fits using the Borda score (calculated from the SCCC, NV-S and SMI scores) are placed accurately within the 20 Å resolution map (shown in grey). The native chain C is shown as reference in yellow [as in (*a*)].

We use *TEMPy* to assess the outcome of a global rigid fitting of a protein into the density map of a complex (a typical scenario, especially when the atomic structure of some of the other components is unknown). Several automated rigid-body fitting programs are available to globally fit either single or multiple component structures into EM density maps (Villa & Lasker, 2014 ▸). Assessing the accuracy of the placement of the components given as the top-ranking solutions by these programs is fundamental to gain insight into the native configuration of the multicomponent system. Here we use the X-ray structure of the Arp2/3 seven-subunit complex with ATP and Ca^2+^ (PDB code 1tyq; Nolen *et al.*, 2004 ▸) as a test case (Fig. 4 ▸ *a*). A 20 Å resolution simulated map was obtained using *Chimera* as described above. A global search of the seven-bladed beta-propeller ARCP1 subunit (chain C) was performed automatically using *ADP_EM* (Garzón *et al.*, 2007 ▸) without any *a priori* assumptions. The top-ten solutions ranked by *ADP_EM* (out of 100) placed chain C in two distinct areas of the EM density map (Fig. 4 ▸ *b*). To examine if a clearer solution can be identified, we re-ranked the entire *ADP_EM* ensemble using the SCCC, NV-S and SMI scores and hierarchically clustered it by Cα-RMSD (data not shown). According to our ranking, the majority of the top-scoring fits belong to the same cluster, suggesting that it may represent the correct placement of the subunit.

Additionally, we applied the consensus scoring to the global ensemble. Calculating the Borda score confirmed that the top-ranked model obtained with *ADP_EM* is the one that stands out more. Visual inspection of the re-ranked ten top-scoring fits (Fig. 4 ▸ *c*) revealed that all of them are placed accurately within the same region of the map (with the centre of mass within 4.9 Å of that of the native structure). Thus, re-ranking with *TEMPy* helped to identify the correct placement of the protein in the map by selecting near-native solutions from an ensemble of solutions (*i.e.* reduce overfitting).

### 3.2. Local assessment of structure segments in single fits   

Local assessment of structure segments of a single fit can provide a better way to evaluate specific regions of a single model in different scenarios (Atherton *et al.*, 2014 ▸; Pandurangan *et al.*, 2014 ▸). This capability of *TEMPy* can be useful in the context of flexible fitting. To demonstrate this, we present two examples using six simulated maps (Example 1) and one experimental (Example 2). We show how to assess the quality of individual secondary structure elements before, after and during the refinement procedure. In each case, as before, we have used the basic functionalities of *TEMPy*, with the Scoring Function module and the Plotting module for assessment. The snippet of code in Fig. S1B shows how to select a set of individual secondary structure elements from a single fitted model, score them with SCCC and generate *Chimera* attribute files (more detailed examples are available online in the *TEMPy* documentation).

#### 3.2.1. Example 1: simulated benchmark   

We assessed three different test cases comprising a protein in two conformations within maps at two different resolutions (5 and 10 Å). The assessment was performed on previously calculated fitted models resulting from flexible fitting by *Flex-EM*/*RIBFIND* (Pandurangan & Topf, 2012 ▸) of (i) the ligand-bound conformation of the glutamine-binding protein (PDB code 1wdn; Sun *et al.*, 1998 ▸) in the density maps simulated from the ligand-free conformation (PDB code 1ggg); (ii) the ligand-free maltodextrin binding protein (PDB code 1omp; Sharff *et al.*, 1992 ▸) in the maps simulated from the ligand-bound conformation (PDB code 1anf); and (iii) the ligand-bound d-ribose-binding protein (PDB code 2dri; Björkman *et al.*, 1994 ▸) in the maps simulated from the ligand-free conformation (PDB code 1urp). Here, we used the SCCC score to assess the fit quality of individual secondary structure elements (as determined by DSSP; Kabsch & Sander, 1983 ▸) of the initial and final models (Fig. S4). As previously shown, this analysis is useful in finding the consensus between multiple flexible fitting methods and thus can help to identify regions with high variability (that may result from overfitting or local errors in the starting models) (Pandurangan *et al.*, 2014 ▸). Furthermore, using SCCC we examined the quality of the fit after each *Flex-EM* simulated annealing cycle of the refinement procedure (Topf *et al.*, 2008 ▸) (Fig. S5). Visualizing the progression of the refinement process in this manner can help in detecting which regions of the structure are more dynamic.

#### 3.2.2. Example 2: Actin   

**Figure 5:**
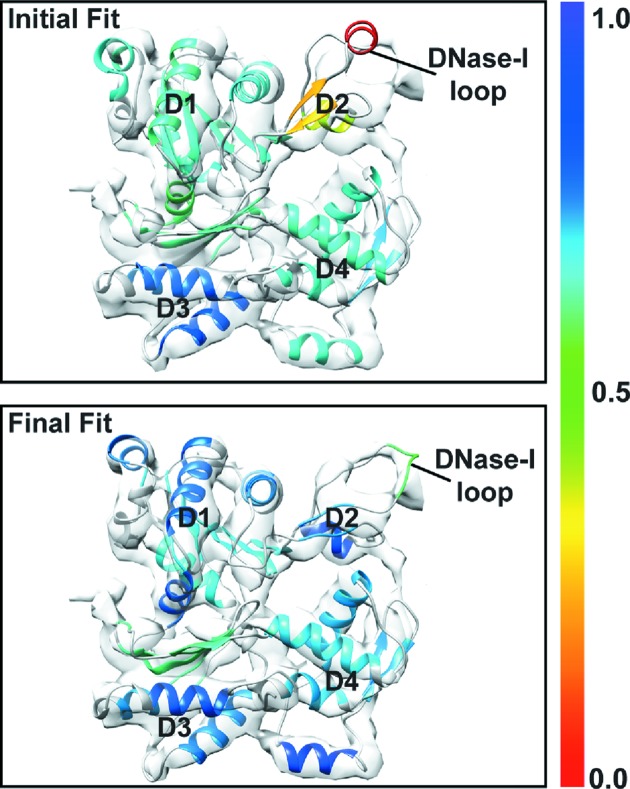
Local assessment of structure segments after refinement of a single fit. Comparison between an actin monomer (initial fit; PDB code 1j6z; Otterbein *et al.*, 2001 ▸) rigidly fitted in an actin monomer density map (segmented from 6.6 Å resolution actin filament; EMD code 5168; Fujii *et al.*, 2010 ▸) and the final model resulting from flexible fitting in the same map (final fit; PDB code 3mfp; Fujii *et al.*, 2010 ▸). Both models are shown within the actin monomer density (in grey). The two models are colour coded according to the SCCC score for each individual secondary structure element (as defined by DSSP). Subdomains 1–4 (D1, D2, D3 and D4, respectively) and the DNase-I loop are labelled.

We also compared, using the segment-based assessment of a single fit, two models: (i) the published model of the F-actin subunit (PDB code 3mfp) refined in the 6.6 Å resolution map of actin filament (EMD code 5168; Fujii *et al.*, 2010 ▸) and (ii) the crystal structure of a unbound G-actin monomer in the ADP state (PDB code 1j6z; Otterbein *et al.*, 2001 ▸), which was used as a starting model. The initial model was rigidly fitted into the actin filament map using *Chimera*’s *fit_in_map* tool. Here too we used the SCCC score to assess the fit quality of individual secondary structure elements (as determined by DSSP) of the initial and final models (Fig. 5 ▸). This type of analysis helps to highlight specific regions where the fit has improved more significantly. As discussed above, an actin subunit consists of four subdomains, with D3 being the most similar in most actin crystal structures (Cong *et al.*, 2008 ▸). This feature is clearly captured by our local assessment having similar quality of fit in the initial and final models (Fig. 5 ▸). On the other end, in D1 our analysis captures subtle changes that could have not been observed using a global analysis approach (*i.e.* scoring the entire model).

## 4. Discussion   

*TEMPy* is a modular library and it has been proven useful in assessing density fits in the context of EM reconstructions (Lukoyanova *et al.*, 2015 ▸; Atherton *et al.*, 2014 ▸; Pandurangan *et al.*, 2014 ▸). It offers a number of distinctive features, in particular the use of multiple scores for the comparison and assessment of fits. In this paper, we introduce *TEMPy*’s capability to assess an individual fit or an ensemble of fits with clustering and with multiple and consensus scoring.

With *TEMPy*, the user can generate a random ensemble or load ensembles that were generated with external software (based on local or global searches) in order to select the best-fitting model relative to alternative fits. Depending on the type of ensemble, this can be useful in assessing a fitted model in terms of accuracy and precision. The scoring function can be selected by the user taking into consideration the resolution and the quality of the EM map, as well as the information available about the fitting component(s) (Vasishtan & Topf, 2011 ▸).

The selection of the most appropriate fit from the gamut of alternative solutions could be based on different strategies. One strategy, widely used in computational biology, is to cluster a set of models on the basis of the RMSD values between each pair (Alber *et al.*, 2008 ▸). In *TEMPy* the user can cluster the fits and visualize the clusters and associated scores for the members of each cluster. This could help to identify the set of fits that score high by multiple methods. It has been shown, for example in small-molecule docking, that relying on the concept of consensus scoring schemes can help to balance errors and increase the ranking power within multiple solutions of docking poses (Kitchen *et al.*, 2004 ▸). Such an approach is also supported *via* the use of the Borda score.

Importantly, in cases where there is no clear single solution resulting from these protocols as the observation-to-parameter ratio is too small for a reliable fitting (owing, for example, to poor resolution, map defects, protein dynamics), multiple solutions can be proposed to better represent the experimental data. This approach is common practice in the NMR field, where atomic coordinates are proposed not only for regions that are well defined by the data but also for ‘ill defined’ regions, which correspond to conformational dynamics and/or reflect incompleteness of the restraining data (Montelione *et al.*, 2013 ▸; Havel & Wüthrich, 1985 ▸). Consequently, each member of the NMR ‘ensemble’ represents a single model that is consistent with the experimental data.

The variability of models across the ensemble provides insight into how well defined are different regions of the structure and the map. Using this strategy to represent EM data can help to describe coordinate uncertainty (Sachse *et al.*, 2007 ▸; Goulet *et al.*, 2014 ▸; Lukoyanova *et al.*, 2015 ▸). The selection of the representative models depends on the different fitting scenarios. For example, if the majority of the top-scoring fits belong to the same non-singleton cluster, the fits in the cluster can be selected as the representative ensemble. Alternatively, depending on the nature of the ensemble, sometimes one can follow the common practice from the protein–protein docking field in which the top 10% of the ensemble (or the 10−20 top models) are chosen. It has been shown in protein–protein docking studies that the majority of the scoring functions routinely used in the field yield an acceptable solution in the majority of the ten top-scoring poses within different docking decoys (Moal *et al.*, 2013 ▸).

Finally, *TEMPy* can also help in addressing the problem of overfitting in flexible fitting. Errors arising from overfitting can be reduced by applying constraints (for example, by grouping atoms together into rigid bodies) during the fitting process (Lopéz-Blanco & Chacón, 2013 ▸; Topf *et al.*, 2008 ▸; Trabuco *et al.*, 2008 ▸; Pandurangan & Topf, 2012 ▸; Grubisic *et al.*, 2010 ▸) and can also be detected by means of consensus between multiple flexible fitting methods (Pandurangan *et al.*, 2014 ▸; Ahmed *et al.*, 2012 ▸; Ahmed & Tama, 2013 ▸). *TEMPy*’s local assessment of structure segments is a useful complementary tool to these approaches.

In conclusion, the modular nature of *TEMPy* makes it a unique platform that will help the user in a fair range of expected fitting scenarios in intermediate-to-low resolution maps. An additional advantage is that it includes the use of plots and output files for visualization purposes that can further help the user in analysing and interpreting density fits at various steps of the fitting process.

## 5. Availability   

The stable release of the library is available for download under Public License from http://tempy.ismb.lon.ac.uk/. The *TEMPy* software package includes well organized documentation built with the *Sphinx* Python documentation generator (http://sphinx-doc.org) and a set of sample scripts that demonstrate usage of the package.

## Supplementary Material

Supplementary figures and tables. DOI: 10.1107/S1600576715010092/vg5014sup1.pdf

## Figures and Tables

**Table 1:** Guidelines to the scores currently available in *TEMPy*

Score	Name	Reference	Note
Cross-correlation coefficient	CCC	Roseman (2000 ▸)	
Segment-based CCC	SCCC	Pandurangan *et al.* (2014 ▸)	Useful for comparing specific regions in multiple fits (Pandurangan *et al.*, 2014 ▸; Atherton *et al.*, 2014 ▸; Lukoyanova *et al.*, 2015 ▸).
Laplacian-filtered CCC	LAP	Wriggers Chacn (2001 ▸)	Useful for resolutions worse than 1015.
Mutual information score	MI	Vasishtan Topf (2011 ▸)	
Segment-based MI	SMI		Useful for comparing specific regions in multiple fits.
Envelope score	ENV	Vasishtan Topf (2011 ▸)	Useful at high resolution. Very fast to calculate and therefore useful in screening fits in large assemblies.
Normal vector score	NV	Vasishtan Topf (2011 ▸), Ceulemans Russell (2004 ▸)	Sensitive to edge detection.
Normal vector score with Sobel filter	NV-S		
Chamfer distance	CD	Vasishtan Topf (2011 ▸)	Highly sensitive to edge detection. Not recommended for segmented maps.
