# Supplementary material for: TEMPy: a Python library for assessment of three-dimensional electron microscopy density fits
Source: J Appl Crystallogr. 2015 Jun 27;48(Pt 4):1314–23. doi: 10.1107/S1600576715010092 (PMC4520291; doi:10.1107/S1600576715010092)
Supplement: Supplementary file 1 [file j-48-01314-sup1.pdf]

JOURNAL OF
APPLIED
CRYSTALLOGRAPHY

Volume 48 (2015)

Supporting information for article:

TEMPy: a Python Library for Assessment of 3D Electron Microscopy Density Fits

Irene Farabella, Daven Vasishtan, Agnel-Praveen Joseph, Arun Prasad Pandurangan, Harpal Sahota and Maya Topf

A

```
from TEMPy.StructureParser import PDBParser
from TEMPy.MapParser import MapParser
from TEMPy.StructureBlurrer import StructureBlurrer
from TEMPy.ScoringFunctions import ScoringFunctions
from TEMPy.EnsembleGeneration import  EnsembleGeneration
from TEMPy.Cluster import  Cluster


fit = PDBParser.read_PDB_file(code,'code.pdb')
map_target = MapParser.readMRC('code.mrc')
fits_ensemble = EnsembleGeneration.randomise_structs(fit,20,10,60)

ranked_ensemble = Cluster.rank_fit_ensemble(fits_ensemble, 'Score', resolution, targetMap=map_target.copy())
mxRMSD = Cluster.RMSD_ensemble(ranked_ensemble, fits_ensemble)
cluster_output = Plot.ShowHierarchicalClusterings(ranked_ensemble, mxRMSD,cutoff, name='Cluster')
```

Import TEMPy functionalities

Load structure and map files

Generate a random ensemble of alternative fits

Rank the ensemble of alternative fits with a given score

Calculate the RMSD all-against-all

Plot the hierarchical clusterd ensemble

B

```
from TEMPy.StructureParser import PDBParser
from TEMPy.RigidBodyParser import RBParser
from TEMPy.MapParser import MapParser
from TEMPy.ScoringFunctions import ScoringFunctions
from TEMPy.ShowPlot import Plot

fit = PDBParser.read_PDB_file(code,'code.pdb')
map_target = MapParser.readMRC('code.mrc')
SCCC_list_structure_instance=[]

rigid_body1=[[55,60]]
rigid_body2=[[79,91]]
list_rigid_bodies=[rigid_body1,rigid_body2]
for rigid_bodies in list_rigid_bodies:
        rigidbody_str_break=fit.break_into_segments(rigid_bodies)
        rigidbody_str_sel=fit.combine_SSE_structures(rigidbody_str_break)
        score_SCCC=scorer.SCCC(map_target, resolution, fit,rigidbody_str_sel)
Plot.PrintOutChimeraAttributeFileSCCC_Score(code,SCCC_list_structure_instance, list_rigid_bodies)
```

Import TEMPy functionalities

Load structure and map files

Define the structure segments

Select each structure segment as a structure instance

Score each structure segments

Generate the Chimera attribute file

Figure S1 (A) Snippet of code to identify a fit that stands out among an ensemble of alternative solutions. The code shows how to read a structure instance (pdb file format) and a map instance (mrc file format), generate an ensemble of fits, rank them based on a chosen score, calculate the all-against-all Cα-RMSD, cluster the ensemble based on Cα-RMSD and then visualise the hierarchically-clustered dendrograms of the fits. (B) Snippet of code to assess the quality of individual secondary structure elements of a fit within a map. The code show how to read a structure instance (pdb file format) and a map instance (mrc file format), select a set of individual secondary structure elements from a single fitted model, generate a ‘rigid-bodies’ list, select each segment as a structure instance, score each segment with a segment-based score and generate the corresponding Chimera attribute files.

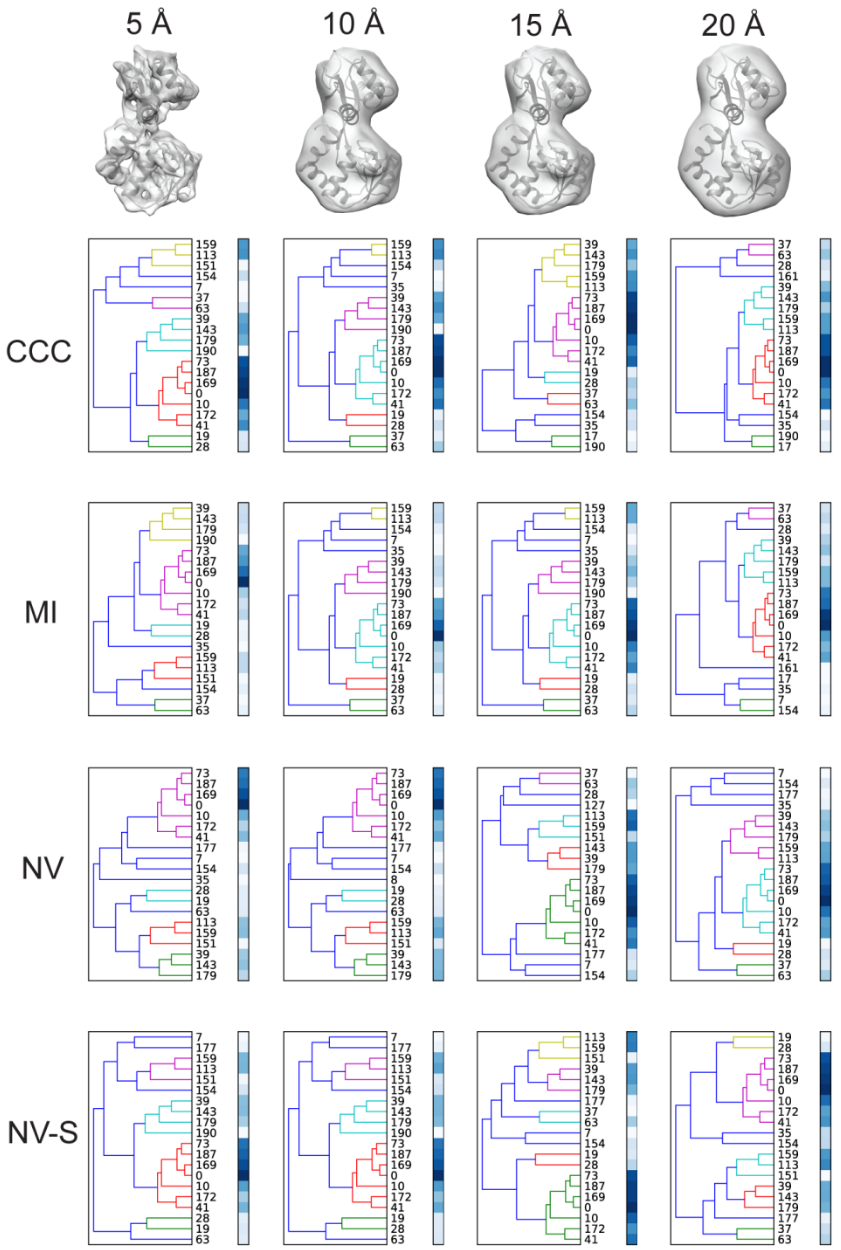

Figure S2 Identification of the top rigid fit of the ligand-free glutamine binding protein [PDB:1ggg (Hsiao *et al.*, 1996)] within the simulated density map at 5, 10, 15, and 20 Å resolution (shown in grey, top raw). Dendrograms representing hierarchical Cα-RMSD clustering of the top 20 fits based on four different scores – CCC (second raw), MI (third raw), NV (fourth raw), and NV with Sobel filter (NV-S, fifth raw) – are shown for each resolution. The colour bars represent the score of each fit from white (lowest score) to blue (highest score) with each cluster coloured differently.

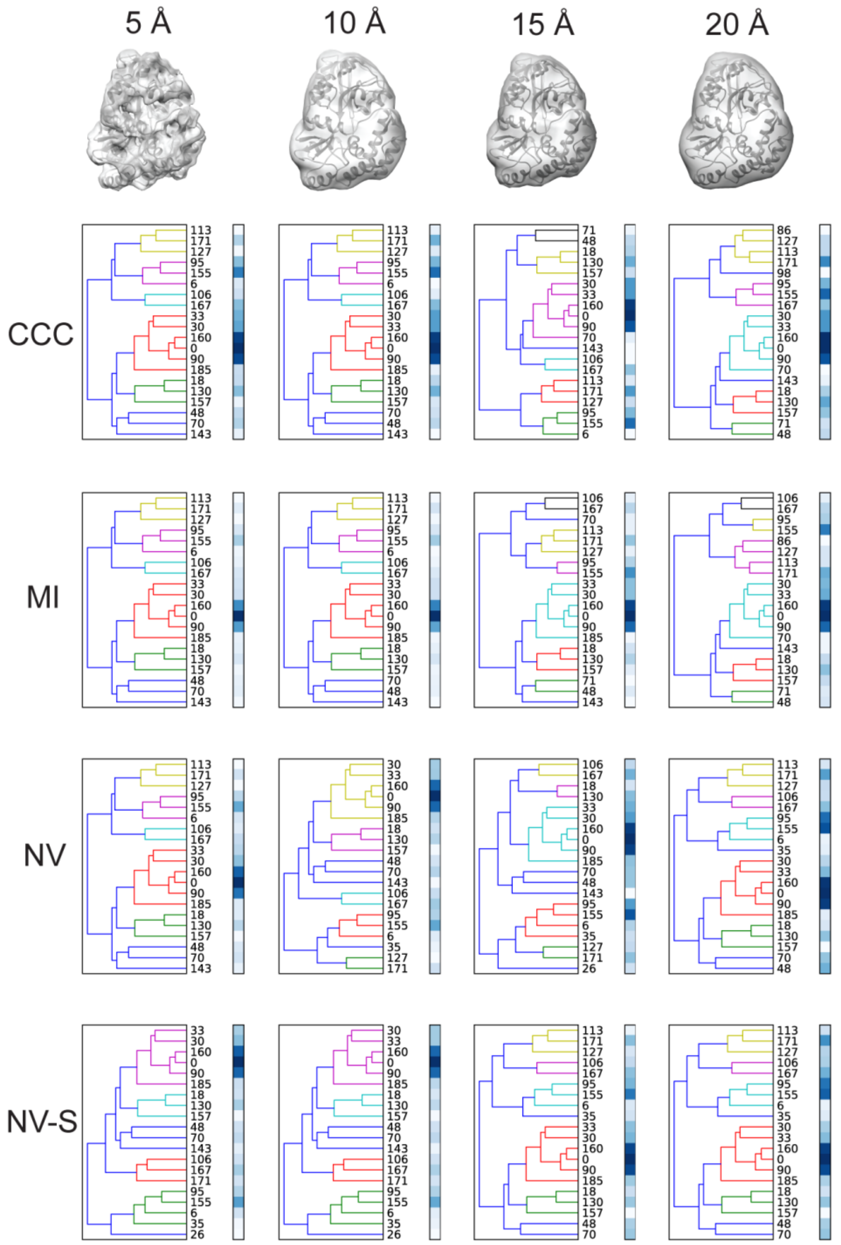

Figure S3 Identification of the top rigid fit of the ligand-bound maltodextrin binding protein [PDB: 1anf; (Quiocho *et al.*, 1997)] within the simulated density map at 5, 10, 15, and 20 Å resolution (shown in grey, top raw). Dendrograms representing hierarchical Cα-RMSD clustering of the top 20 fits based on four different scores – CCC (second raw), MI (third raw), NV (fourth raw), and NV with Sobel filter (NV-S, fifth raw) – are shown for each resolution. The colour bars represent the score of each fit from white (lowest score) to blue (highest score) with each cluster coloured differently.

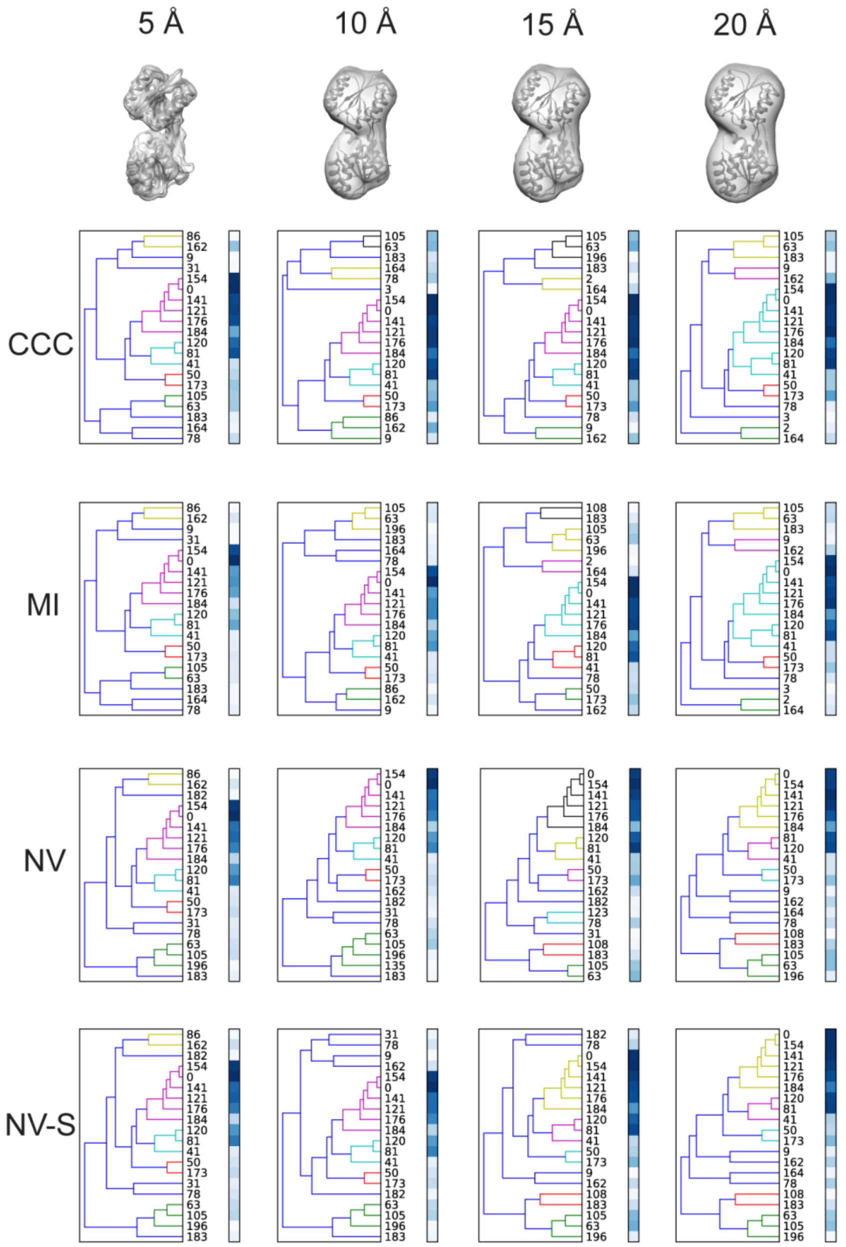

Figure S4 Identification of the top rigid fit of the ligand-free D-ribose-binding protein [PDB: 1urp; (Björkman & Mowbray, 1998)], within the simulated density map at 5, 10, 15, and 20 Å resolution (shown in grey, top raw). Dendrograms representing hierarchical Cα-RMSD clustering of the top 20 fits based on four different scores – CCC (second raw), MI (third raw), NV (fourth raw), and NV with Sobel filter (NV-S, fifth raw) – are shown for each resolution. The colour bars represent the score of each fit from white (lowest score) to blue (best score) with each cluster coloured differently.

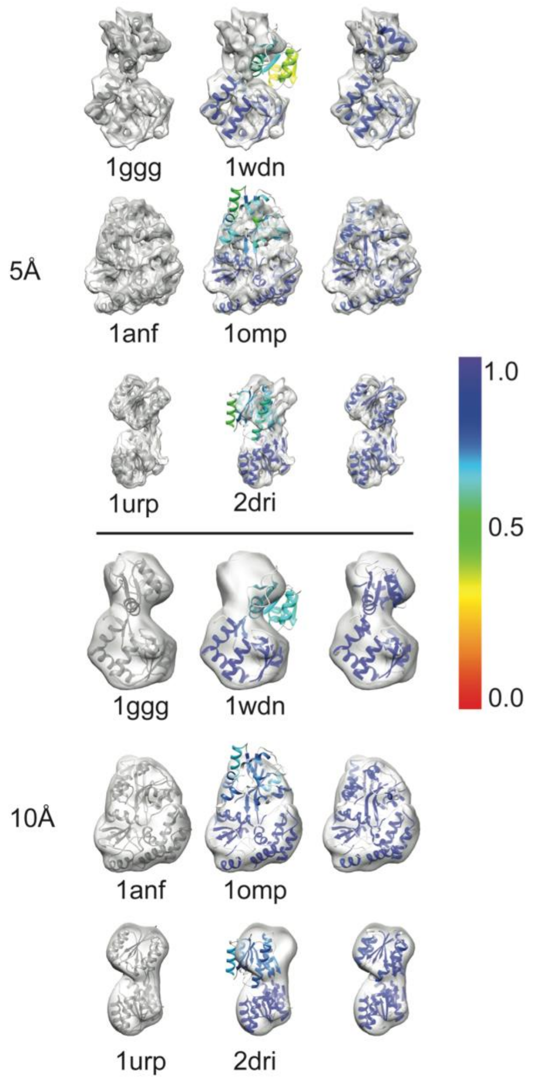

Figure S5 Local assessment of structure segments by comparison of fits before and after refinement using Flex-EM (Pandurangan & Topf, 2012, Topf *et al.*, 2008). Left column: crystal structures (in dark grey) of the ligand-free glutamine binding protein [PDB:1ggg; (Hsiao *et al.*, 1996)], the ligand-bound maltodextrin binding protein [PDB: 1anf; (Quiocho *et al.*, 1997)], and the ligand-free D-ribose-binding protein [PDB: 1urp; (Björkman & Mowbray, 1998)] fitted in the respective simulated maps at 5 and 10 Å resolution (light grey). Centre column: crystal structures of the ligand-bound conformation of the glutamine binding protein [PDB: 1wdn; (Sun *et al.*, 1998)], the ligand-free maltodextrin binding protein [PDB: 1omp; (Sharff *et al.*, 1992))], and the ligand-bound D-ribose-binding protein [PDB: 2dri (Bjorkman *et al.*, 1994)] rigidly fitted in the simulated maps (which are based on the other conformation, as in the left column). The fits are colour-coded according to the SCCC score for each individual secondary structure elements [as defined by DSSP (Kabsch & Sander, 1983)] and the densities are shown within the in light grey. Right column: the final models after refinement with Flex-EM are shown within the respective density maps (as in the left and centre columns, shown in light grey) and colour-coded by SCCC score for each individual secondary structure elements.

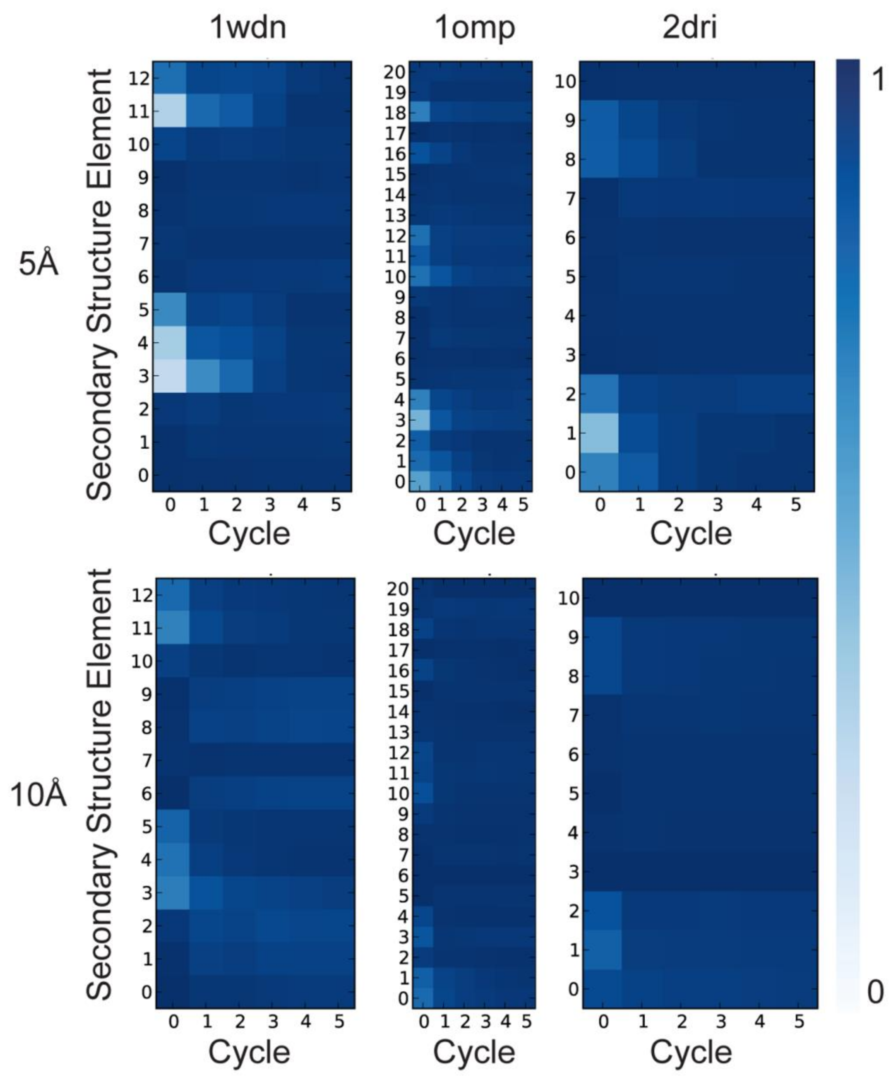

Figure S6 Heat map showing the quality of the fit of individual secondary structure elements [as determined by DSSP (Kabsch & Sander, 1983)] after each of the first five simulated annealing cycles of Flex-EM refinement (Pandurangan & Topf, 2012, Topf *et al.*, 2008) for: the ligand-bound conformation of the glutamine binding protein [PDB: 1wdn; (Sun *et al.*, 1998)] in the density maps simulated from the ligand-free conformation [PDB:1ggg; (Hsiao *et al.*, 1996)]; the ligand-free maltodextrin binding protein [PDB: 1omp; (Sharff *et al.*, 1992))] in the maps simulated from the ligand-bound conformation [PDB: 1anf; (Quiocho *et al.*, 1997)]; and the ligand-bound D-ribose-binding protein [PDB: 2dri (Bjorkman *et al.*, 1994)] in the maps simulated from the ligand-free conformation [PDB: 1urp; (Björkman & Mowbray, 1998)]. The colour (see key) denotes the SCCC score.

Table S1 Consensus score for the 20 top-scoring fits of the ligand-free glutamine binding protein [PDB:1ggg; (Hsiao et al., 1996)] within the simulated density map at 5 Å resolution.

Fit	CCC	MI	NV-S	Borda	Re-Rank
mod_0	1	1	1	597	1
mod_169	2	2	2	594	2
mod_187	3	3	3	591	3
mod_73	4	4	4	588	4
mod_10	5	5	5	585	5
mod_41	6	6	6	582	6
mod_113	7	7	7	579	7
mod_159	8	8	8	576	8
mod_143	9	9	9	573	9
mod_39	10	10	10	570	10
mod_172	11	11	11	567	11
mod_179	12	12	12	564	12
mod_154	13	13	13	561	13
mod_63	14	14	15	557	14
mod_19	16	16	14	554	15
mod_28	15	15	16	554	16
mod_151	17	17	18	548	17
mod_37	18	18	21	543	18
mod_190	19	19	20	542	19
mod_177	21	22	17	540	20

Descriptions of the items are: Fit, the model number; CCC, rank based on the cross-correlation score; MI, rank based on the mutual information score; NV-S, rank based on the normal vector score with Sobel filter; Borda, Borda consensus score; Re-Rank, rank based on the consensus method (Borda).

Table S2 Consensus score for the 20 top-scoring fits of the ligand-free glutamine binding protein [PDB:1ggg; (Hsiao et al., 1996)] within the simulated density map at 10 Å resolution.

Fit	CCC	MI	NV-S	Borda	Re-Rank
mod_0	1	1	1	597	1
mod_169	2	2	2	594	2
mod_187	3	3	3	591	3
mod_73	4	4	4	588	4
mod_10	5	5	5	585	5
mod_41	6	6	7	581	6
mod_113	7	7	6	580	7
mod_143	9	8	9	574	8
mod_159	10	10	8	572	9
mod_172	8	9	12	571	10
mod_39	11	11	10	568	11
mod_179	12	12	11	565	12
mod_63	13	13	15	559	13
mod_154	14	14	13	559	14
mod_28	15	15	16	554	15
mod_19	17	16	17	550	16
mod_151	21	21	14	544	17
mod_37	16	17	24	543	18
mod_190	19	19	20	542	19
mod_7	20	20	19	541	20

Descriptions of the items are: Fit, the model number; CCC, rank based on the cross-correlation score; MI, rank based on the mutual information score; NV-S, rank based on the normal vector score with Sobel filter; Borda, Borda consensus score; Re-Rank, rank based on the consensus method (Borda).

Table S3 Consensus score for the 20 top-scoring fits of the ligand-free glutamine binding protein [PDB:1ggg; (Hsiao et al., 1996)] within the simulated density map at 15 Å resolution.

Fit	CCC	MI	NV-S	Borda	Re-Rank
mod_0	1	1	1	597	1
mod_169	2	2	2	594	2
mod_187	3	3	3	591	3
mod_73	4	4	4	588	4
mod_10	5	5	5	585	5
mod_41	6	6	6	582	6
mod_172	7	7	9	577	7
mod_113	8	8	8	576	8
mod_159	10	9	7	574	9
mod_143	9	10	10	571	10
mod_39	11	11	11	567	11
mod_179	13	12	12	563	12
mod_63	12	13	13	562	13
mod_28	14	14	14	558	14
mod_154	17	17	15	551	15
mod_37	15	16	20	549	16
mod_19	18	18	16	548	17
mod_35	16	15	22	547	18
mod_190	19	19	21	541	19
mod_7	21	20	19	540	20

Descriptions of the items are: Fit, the model number; CCC, rank based on the cross-correlation score; MI, rank based on the mutual information score; NV-S, rank based on the normal vector score with Sobel filter; Borda, Borda consensus score; Re-Rank, rank based on the consensus method (Borda).

Table S4 Consensus score for the 20 top-scoring fits of the ligand-free glutamine binding protein [PDB:1ggg; (Hsiao et al., 1996)] within the simulated density map at 20 Å resolution.

Fit	CCC	MI	NV-S	Borda	Re-Rank
mod_0	1	1	1	597	1
mod_169	2	2	2	594	2
mod_187	3	3	3	591	3
mod_73	4	4	4	588	4
mod_10	6	5	5	584	5
mod_41	5	6	6	583	6
mod_113	9	7	7	577	7
mod_172	8	9	8	575	8
mod_143	7	10	10	573	9
mod_159	10	8	9	573	10
mod_39	11	11	11	567	11
mod_63	12	12	12	564	12
mod_179	13	13	13	561	13
mod_37	14	14	16	556	14
mod_28	16	15	14	555	15
mod_35	15	16	17	552	16
mod_154	19	17	15	549	17
mod_17	18	19	24	539	18
mod_161	17	18	26	539	19
mod_7	21	20	20	539	20

Descriptions of the items are: Fit, the model number; CCC, rank based on the cross-correlation score; MI, rank based on the mutual information score; NV-S, rank based on the normal vector score with Sobel filter; Borda, Borda consensus score; Re-Rank, rank based on the consensus method (Borda).

Table S5 Consensus score for the 20 top-scoring fits of the ligand-bound maltodextrin binding protein [PDB: 1anf; (Quiocho et al., 1997)] within the simulated density map at 5 Å resolution.

Fit	CCC	MI	NV-S	Borda	Re-Rank
mod_0	1	1	1	597	1
mod_160	2	2	2	594	2
mod_90	3	3	3	591	3
mod_155	4	4	4	588	4
mod_30	5	5	5	585	5
mod_33	6	6	7	581	6
mod_130	7	7	8	578	7
mod_167	8	9	6	577	8
mod_95	9	8	9	574	9
mod_70	11	11	10	568	10
mod_171	10	10	15	565	11
mod_18	13	12	12	563	12
mod_185	14	14	11	561	13
mod_48	12	13	14	561	14
mod_106	15	15	13	557	15
mod_6	16	16	16	552	16
mod_143	17	18	17	548	17
mod_157	18	17	19	546	18
mod_127	19	19	22	540	19
mod_113	20	20	21	539	20

Descriptions of the items are: Fit, the model number; CCC, rank based on the cross-correlation score; MI, rank based on the mutual information score; NV-S, rank based on the normal vector score with Sobel filter; Borda, Borda consensus score; Re-Rank, rank based on the consensus method (Borda).

Table S6 Consensus score for the 20 top-scoring fits of the ligand-bound maltodextrin binding protein [PDB: 1anf; (Quiocho et al., 1997)] within the simulated density map at 10 Å resolution.

Fit	CCC	MI	NV-S	Borda	Re-Rank
mod_0	1	1	1	597	1
mod_160	2	2	3	593	2
mod_90	3	3	2	592	3
mod_155	4	4	4	588	4
mod_33	5	5	5	585	5
mod_30	6	6	6	582	6
mod_95	9	9	8	574	7
mod_130	8	8	11	573	8
mod_167	10	10	7	573	9
mod_171	7	7	14	572	10
mod_70	13	13	10	564	11
mod_18	11	11	15	563	12
mod_48	12	12	13	563	13
mod_185	17	16	9	558	14
mod_106	16	15	12	557	15
mod_157	14	14	19	553	16
mod_127	15	17	22	546	17
mod_6	20	19	16	545	18
mod_143	19	20	18	543	19
mod_113	18	18	24	540	20

Descriptions of the items are: Fit, the model number; CCC, rank based on the cross-correlation score; MI, rank based on the mutual information score; NV-S, rank based on the normal vector score with Sobel filter; Borda, Borda consensus score; Re-Rank, rank based on the consensus method (Borda).

Table S7 Consensus score for the 20 top-scoring fits of the ligand-bound maltodextrin binding protein [PDB: 1anf; (Quiocho et al., 1997)] within the simulated density map at 15 Å resolution.

Fit	CCC	MI	NV-S	Borda	Re-Rank
mod_0	1	1	1	597	1
mod_160	2	2	2	594	2
mod_90	3	3	3	591	3
mod_155	4	4	4	588	4
mod_30	6	5	5	584	5
mod_33	5	6	9	580	6
mod_171	7	7	7	579	7
mod_95	9	9	6	576	8
mod_130	8	8	11	573	9
mod_167	10	10	8	572	10
mod_18	11	11	15	563	11
mod_48	12	12	13	563	12
mod_70	16	14	10	560	13
mod_157	13	13	20	554	14
mod_127	14	17	17	552	15
mod_106	19	15	14	552	16
mod_113	15	16	19	550	17
mod_185	21	18	12	549	18
mod_143	18	19	22	541	19
mod_6	20	21	18	541	20

Descriptions of the items are: Fit, the model number; CCC, rank based on the cross-correlation score; MI, rank based on the mutual information score; NV-S, rank based on the normal vector score with Sobel filter; Borda, Borda consensus score; Re-Rank, rank based on the consensus method (Borda).

Table S8 Consensus score for the 20 top-scoring fits of the ligand-bound maltodextrin binding protein [PDB: 1anf; (Quiocho et al., 1997)] within the simulated density map at 20 Å resolution.

Fit	CCC	MI	NV-S	Borda	Re-Rank
mod_0	1	1	1	597	1
mod_160	2	2	2	594	2
mod_90	3	3	3	591	3
mod_155	4	4	4	588	4
mod_171	5	5	6	584	5
mod_33	6	6	8	580	6
mod_95	9	9	5	577	7
mod_130	8	8	10	574	8
mod_30	7	7	12	574	9
mod_167	10	10	7	573	10
mod_18	12	11	17	560	11
mod_48	15	15	11	559	12
mod_127	13	14	15	558	13
mod_157	11	12	20	557	14
mod_113	14	13	16	557	15
mod_70	18	19	9	554	16
mod_106	22	17	14	547	17
mod_71	16	16	25	543	18
mod_143	17	18	22	543	19
mod_185	23	22	13	542	20

Descriptions of the items are: Fit, the model number; CCC, rank based on the cross-correlation score; MI, rank based on the mutual information score; NV-S, rank based on the normal vector score with Sobel filter; Borda, Borda consensus score; Re-Rank, rank based on the consensus method (Borda).

Table S9 Consensus score for the 20 top-scoring fits of the ligand-free D-ribose-binding protein [PDB: 1urp; (Björkman & Mowbray, 1998)] within the simulated density map at 5 Å resolution.

Fit	CCC	MI	NV-S	Borda	Re-Rank
mod_0	1	1	1	588	1
mod_154	2	2	2	585	2
mod_121	3	3	3	582	3
mod_141	4	4	4	579	4
mod_176	5	5	6	575	5
mod_81	6	6	5	574	6
mod_120	7	7	7	570	7
mod_184	8	8	9	566	8
mod_162	9	9	12	561	9
mod_173	10	10	11	560	10
mod_63	11	11	10	559	11
mod_105	12	12	8	559	12
mod_50	13	13	13	552	13
mod_78	14	15	14	548	14
mod_41	15	14	15	547	15
mod_183	16	16	17	542	16
mod_31	18	19	16	538	17
mod_86	19	18	19	535	18
mod_164	17	17	24	533	19
mod_9	20	20	22	529	20

Descriptions of the items are: Fit, the model number; CCC, rank based on the cross-correlation score; MI, rank based on the mutual information score; NV-S, rank based on the normal vector score with Sobel filter; Borda, Borda consensus score; Re-Rank, rank based on the consensus method (Borda).

Table S10 Consensus score for the 20 top-scoring fits of the ligand-free D-ribose-binding protein [PDB: 1urp; (Björkman & Mowbray, 1998)] within the simulated density map at 10 Å resolution.

Fit	CCC	MI	NV-S	Borda	Re-Rank
mod_0	1	1	1	588	1
mod_154	2	2	2	585	2
mod_121	4	4	3	580	3
mod_176	3	3	6	579	4
mod_81	5	5	5	576	5
mod_141	6	6	4	575	6
mod_120	7	7	7	570	7
mod_184	8	8	9	566	8
mod_173	9	9	11	562	9
mod_63	11	11	10	559	10
mod_162	10	10	12	559	11
mod_105	13	12	8	558	12
mod_50	12	14	13	552	13
mod_41	14	13	15	549	14
mod_78	15	15	14	547	15
mod_183	17	17	17	540	16
mod_9	18	18	20	535	17
mod_164	16	16	25	534	18
mod_86	19	19	21	532	19
mod_196	21	20	19	531	20

Descriptions of the items are: Fit, the model number; CCC, rank based on the cross-correlation score; MI, rank based on the mutual information score; NV-S, rank based on the normal vector score with Sobel filter;Borda, Borda consensus score; Re-Rank, rank based on the consensus method (Borda).

Table S11 Consensus score for the 20 top-scoring fits of the ligand-free D-ribose-binding protein [PDB: 1urp; (Björkman & Mowbray, 1998)] within the simulated density map at 15 Å resolution.

Fit	CCC	MI	NV-S	Borda	Re-Rank
mod_0	1	1	2	587	1
mod_154	2	2	1	586	2
mod_176	3	3	6	579	3
mod_121	4	4	5	578	4
mod_141	5	5	3	578	5
mod_81	6	6	4	575	6
mod_120	7	7	7	570	7
mod_184	8	8	8	567	8
mod_173	9	9	10	563	9
mod_63	10	10	9	562	10
mod_105	11	11	11	558	11
mod_50	14	13	12	552	12
mod_162	13	12	15	551	13
mod_41	12	14	14	551	14
mod_78	16	15	13	547	15
mod_183	17	17	16	541	16
mod_164	15	16	22	538	17
mod_196	20	18	18	535	18
mod_9	18	21	20	532	19
mod_108	22	19	19	531	20

Descriptions of the items are: Fit, the model number; CCC, rank based on the cross-correlation score; MI, rank based on the mutual information score; NV-S, rank based on the normal vector score with Sobel filter;Borda, Borda consensus score; Re-Rank, rank based on the consensus method (Borda).

Table S12 Consensus score for the 20 top-scoring fits of the ligand-free D-ribose-binding protein [PDB: 1urp; (Björkman & Mowbray, 1998)] within the simulated density map at 20 Å resolution.

Fit	CCC	MI	NV-S	Borda	Re-Rank
mod_0	1	1	2	587	1
mod_154	2	2	1	586	2
mod_121	4	4	4	579	3
mod_141	5	5	3	578	4
mod_176	3	3	7	578	5
mod_81	6	6	5	574	6
mod_120	7	7	6	571	7
mod_184	8	8	8	567	8
mod_173	9	9	9	564	9
mod_63	11	11	11	558	10
mod_162	10	10	13	558	11
mod_105	14	12	10	555	12
mod_41	12	13	14	552	13
mod_50	13	14	15	549	14
mod_78	17	16	12	546	15
mod_164	15	15	18	543	16
mod_183	18	17	16	540	17
mod_9	19	19	17	536	18
mod_196	21	21	19	530	19
mod_2	16	18	28	529	20

Descriptions of the items are: Fit, the model number; CCC, rank based on the cross-correlation score; MI, rank based on the mutual information score; NV-S, rank based on the normal vector score with Sobel filter;Borda, Borda consensus score; Re-Rank, rank based on the consensus method (Borda).

Table S13 Consensus score for the 20 top-scoring fits of a single GroEL subunit [PDB: 1oel; (Braig et al., 1995)] within the 11.5 Å resolution density map of GroEL [EMD: 1080; (Ludtke *et al.*, 2001)].

Fit	CCC	MI	NV-S	Borda	Re-Rank
mod_0	1	1	1	2994	1
mod_197	5	3	2	2987	2
mod_755	2	2	6	2987	3
mod_259	4	6	5	2982	4
mod_118	6	5	4	2982	5
mod_133	7	7	3	2980	6
mod_774	3	4	14	2976	7
mod_241	8	14	8	2967	8
mod_565	9	10	12	2966	9
mod_509	12	9	11	2965	10
mod_1	13	13	7	2964	11
mod_415	11	8	16	2962	12
mod_763	14	11	10	2962	13
mod_457	10	16	9	2962	14
mod_756	16	12	15	2954	15
mod_881	15	17	13	2952	16
mod_969	17	15	17	2948	17
mod_994	18	19	18	2942	18
mod_935	20	18	19	2940	19
mod_914	19	20	20	2938	20

Descriptions of the items are: Fit, the model number; CCC, rank based on the cross-correlation score; MI, rank based on the mutual information score; NV-S, rank based on the normal vector score with Sobel filter; Borda, Borda consensus score ; Re-Rank, rank based on the consensus method (Borda).
